# Understanding Auditory Spectro-Temporal Receptive Fields and Their Changes with Input Statistics by Efficient Coding Principles

**DOI:** 10.1371/journal.pcbi.1002123

**Published:** 2011-08-18

**Authors:** Lingyun Zhao, Li Zhaoping

**Affiliations:** 1 Department of Biomedical Engineering, School of Medicine, Tsinghua University, Beijing, P.R. China; 2 Department of Computer Science, University College London, London, United Kingdom; Université Paris Descartes, Centre National de la Recherche Scientifique, France

## Abstract

Spectro-temporal receptive fields (STRFs) have been widely used as linear approximations to the signal transform from sound spectrograms to neural responses along the auditory pathway. Their dependence on statistical attributes of the stimuli, such as sound intensity, is usually explained by nonlinear mechanisms and models. Here, we apply an efficient coding principle which has been successfully used to understand receptive fields in early stages of visual processing, in order to provide a computational understanding of the STRFs. According to this principle, STRFs result from an optimal tradeoff between maximizing the sensory information the brain receives, and minimizing the cost of the neural activities required to represent and transmit this information. Both terms depend on the statistical properties of the sensory inputs and the noise that corrupts them. The STRFs should therefore depend on the input power spectrum and the signal-to-noise ratio, which is assumed to increase with input intensity. We analytically derive the optimal STRFs when signal and noise are approximated as Gaussians. Under the constraint that they should be spectro-temporally local, the STRFs are predicted to adapt from being band-pass to low-pass filters as the input intensity reduces, or the input correlation becomes longer range in sound frequency or time. These predictions qualitatively match physiological observations. Our prediction as to how the STRFs should be determined by the input power spectrum could readily be tested, since this spectrum depends on the stimulus ensemble. The potentials and limitations of the efficient coding principle are discussed.

## Author Summary

Spectro-temporal receptive fields (STRFs) have been widely used as linear approximations of the signal transform from sound spectrograms to neural responses along the auditory pathway. Their dependence on the ensemble of input stimuli has usually been examined mechanistically as a possibly complex nonlinear process. We propose that the STRFs and their dependence on the input ensemble can be understood by an efficient coding principle, according to which the responses of the encoding neurons report the maximum amount of information about the sensory input, subject to limits on the neural cost in representing and transmitting information. This proposal is inspired by the success of the same principle in accounting for receptive fields in the early stages of the visual pathway and their adaptation to input statistics. The principle can account for the STRFs that have been observed, and the way they change with sound intensity. Further, it predicts how the STRFs should change with input correlations, an issue that has not been extensively investigated. In sum, our study provides a computational understanding of the neural transformations of auditory inputs, and makes testable predictions for future experiments.

## Introduction

In response to acoustic input signals, neurons in the auditory pathway are typically selective to sound frequency 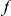 and have particular response latencies. At least ignoring cases with 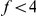 kHz, in which neuronal responses often phase lock to the sound waves, a spectro-temporal receptive field (STRF) is often used to describe the tuning properties of a neuron [1], [2], [3], [4]. This is a two-dimensional function 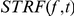 that reports the sensitivity of the neuron at response latency 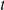 to acoustic inputs of frequency 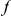 for a given stimulus ensemble (i.e., given input statistics). More specifically, in a stimulus ensemble, the power 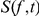 of the acoustic input at frequency 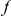 at time 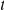 fluctuates around an average level denoted by 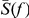. If we let 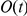 denote the neuron's response at time 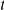 (typically its spike rate), then 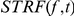 best approximates the linear relationship between 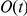 and 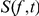 in this stimulus ensemble as
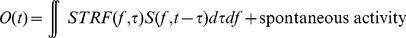
(1)Note that in this paper, we refer to 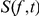 as the input spectrogram, although some authors also include the average input power 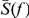. Though 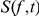 is not a full description of acoustic input, since it ignores features such as the phase of the oscillation in the sound wave, it is the only relevant aspect of the auditory input as far as the STRF is concerned. Note that if we use 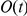 to denote the deviation of the neural response from its spontaneous activity level, then both 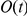 and 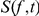 have zero mean. We will use this simplification throughout the paper. In studies in which the temporal dimension is omitted, the STRF is called the spectral receptive field (SRF).

**Figure 1:**
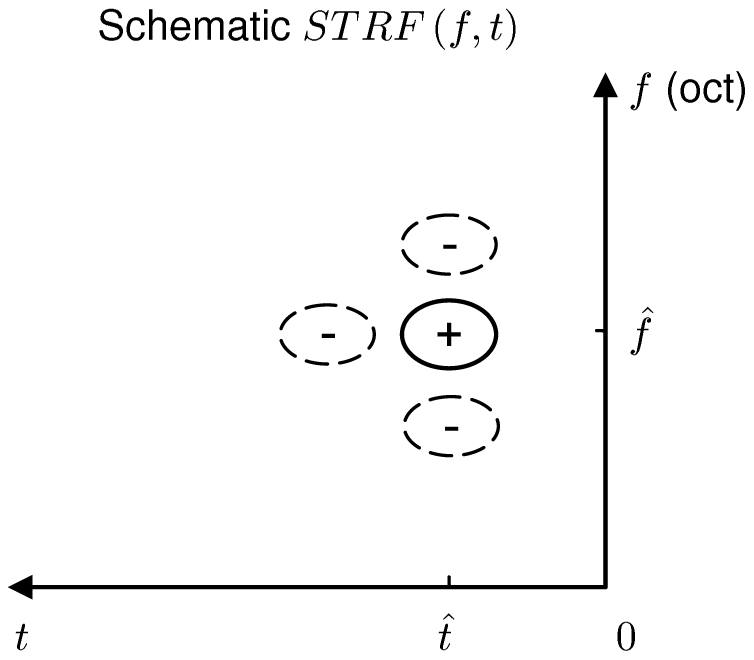
A schematic example of a typical spectro-temporal receptive field, plotted with a reversed abscissa. This STRF has one excitatory and three inhibitory regions, prefers frequency 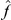, and evokes response at a typical latency 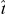. Since the response at time 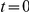 is 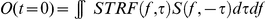, an input stimulus 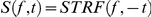 exactly as depicted in this plot is most likely to elicit a large response 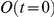 at time 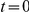, or indeed a spike.

Figure 1 cartoons a typical STRF. This has excitatory and inhibitory regions, reflecting its preferred frequency and response latency. For example, if 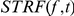 peaks at frequency 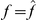 and time 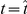, then this neuron prefers frequency 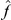 and should respond to an input impulse 
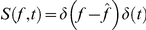
 of this frequency with latency 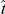. We will also refer to 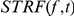 as the receptive field, the filter kernel, or the transfer function from input to neural responses, as these all convey the same or similar meanings. A neuron's STRF is typically estimated using reverse correlation methods [5], [4].

However, there are extensive nonlinearities in the signal transformation along the auditory pathway. Indeed, the STRF formulation of neural responses, though linear in spectral power, is already a second-order nonlinear function of the auditory sound wave. There are two kinds of nonlinearities when inputs are represented as spectrograms. The simpler one is a static nonlinearity 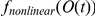, which when applied to the linear approximation 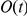 of equation (1) enables better predictions of the neural responses [6], [7]. This static nonlinearity however does not alter the spectro-temporal selectivity of the neuron seen in the linear STRF. This paper is interested in the more complex nonlinearity that the STRFs are dependent on the stimulus ensemble used to estimate them [1], [5], [8], [9]. For example, the STRFs are wider when the stimuli are narrow-band rather than wide-band [10], or when the stimuli are animal vocalizations rather than noise [11]. The STRF (or SRF) also becomes more band-pass when sound intensity increases. The dependence of the STRFs on the stimulus ensemble holds, for example, for type IV neurons in the cochlear nucleus of cats [12], [13], the inferior colliculus (IC) of the frog [8] and the gerbil [7], and field L region of the songbird (which is analogous to mammalian auditory cortex) [14]. (The dependence on sound intensity also holds for the linear relationship between the auditory nerve responses and input sound waves [5]). Nonlinearities in the auditory system become progressively stronger further from the periphery.

Despite the nonlinearities, the concept of the STRF is still widely used, not only because it provides a meaningful description of the spectro-temporal selectivity of the neurons in a given stimulus ensemble, but also because it can predict neural responses to novel stimuli reasonably well, as long as the stimuli are drawn from the same stimulus ensemble as that used to estimate the STRF in the first place. Reasonable predictions from the STRFs have been obtained for the responses of auditory nerves(see [15]) and auditory midbrain neurons [6], [7], [16] (also see [2]). They have also been obtained for responses of the auditory cortical neurons when the stimulus ensemble is composed of biologically more meaningful static or dynamic ripples (broadband sound with sinusoidally modulated spectral envelopes and their linear combinations [17], [18], [19]). If the linear neural filter is augmented to include the filtering performed by the head and ears, it is also possible to predict the preferred locations of sound sources of auditory cortical neurons based on the linear neural filter for input spectrograms [20]. Meanwhile, linear STRF models fail to capture many complex phenomena, particularly in the auditory cortex, and nonlinearities are not limited to being just static or monotonic. It has been suggested that some auditory cortical neurons process auditory objects in a highly non-linear manner, by selectively responding to a weak object component while ignoring loud components that occupy the same region in frequency space in auditory mixtures of these object components [21], and some prefer low over high spectral contrast sounds [22]. Strong nonlinearities in the auditory processes have long since motivated nonlinear models of auditory responses (e.g., [5], [12], [23]).

This paper aims to understand from a computational, rather than a mechanistic, perspective why the auditory encoding transform should depend on the stimulus ensemble in the ways observed. More specifically, the paper focuses on cases in which STRFs can reasonably capture neural responses, and aims to identify and understand the computational goal of the STRFs for a given stimulus ensemble – finding a metric according to which the STRFs are optimal for the ensemble. This would provide a rationale for how the physiologically measured STRFs should depend on or adapt to the stimulus ensemble. This paper does not address what linear or nonlinear mechanisms could build the optimal STRFs, or whether or how nonlinear auditory processes enable the adaptation of the STRFs to the stimulus ensemble. Existing computational models of auditory neurons, including ones with the notion that cochlear hair cells perform independent component analysis to provide an efficient code for inputs using spikes in the auditory nerves [24], [25], cannot explain the observed dependence of the STRFs on the stimulus ensemble (see Discussion for more details).

Restricting attention to the temporal properties of STRF, Lesica and Grothe [26] observed that the temporal filter in STRF adapted to the level of ambient noise in the input environment. In particular, the temporal receptive field in the STRF changed from being bandpass to being low pass with the increase of ambient noise. They argued using a simple model that such adaptation in the STRF enables more efficient coding of the input information.

This study applies the principles of efficient coding to understand the auditory STRF and its variations with sound intensities and other input characteristics. It generalizes the work of Lesica and Grothe [26] to understand the temporal and spectral filtering characteristics of STRF adaptation to changes in noise, signal and correlations in input statistics. Explicitly, the principle of efficient coding states that the neural receptive fields should enable the neural responses to transmit as much sensory information as possible to the central nervous system, subject to the limitation in neural cost in representing and transmitting information. This principle has been proposed [27] and successfully applied to the visual system to understand the receptive fields in the early visual pathway [28], [29], [30], [31], [32], [33] (see review [34]). We will borrow heavily techniques and intuitions from vision to derive and explain the results in this paper.

To make initial progress, it is necessary to start with some simplifying assumptions. First, we assume that the statistical characteristics of the stimulus ensemble do not change more rapidly than the speed at which the sensory encoding adapts, so that the stimulus ensemble can be approximated as being stationary as far as optimal encoding is concerned. Knowing when this assumption does not hold tells us when the encoding is not optimal, e.g., when one sees poorly for a brief moment before the visual encoding adapts to a sudden change from a dark room to a bright garden. Second, for mathematical convenience, we assume that the linear STRF model as in equation (1) can approximate adapted auditory neural responses reasonably well. As we know from above, this assumption often does not hold, particularly for auditory cortical neurons. This paper leaves the extension of the optimal encoding to nonlinear cases for future studies. Third, to derive a closed-form, analytical, solution to the optimal STRF, we assume that the input statistics in the stimulus ensemble can be approximated as being Gaussian, with higher order correlations in the input contributing only negligibly to the inefficiency of the representation in the original sensory inputs. Although it is known that the natural auditory inputs are far from Gaussian [35], as for the case of vision, the discrepancy may have only a limited impact on the input inefficiency, as measured by the amount of information redundancy in the original sensory input [36], [37], [38].

To understand how sensory inputs should be recoded to increase coding efficiency, we start with visual encoding to draw insights and made analogies with auditory encoding. In vision, large amounts of raw data about the visual world are transduced by photoreceptors. However, the optic nerve, which transmits the input data to the visual cortex via thalamus, can only accommodate a dramatically smaller data rate. It has thus been proposed that early visual processes use an efficient coding strategy to encode as much information as possible given the limited bandwidth [27], [34], in other words, to recode the data such that the redundancy in the data is reduced and consequently the data can be transmitted by the limited bandwidth. Compression (while preserving most information) is possible since images are very redundant [39], [40], [41], [42], e.g., with strong correlations between visual inputs at nearby points in time and space. Removing such correlations can cut down the data rate substantially [34].

One way to remove the correlations is to transform the raw input 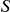 into a different representation 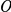 in neural responses that would then have a much smaller data rate than 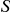, yet preserving essential input information. This transform is often approximated by the visual receptive field, analogous to the auditory STRFs. For instance, the (spatial) center-surround receptive fields of the retinal ganglion cells help remove spatial redundancy [30], [31], [43]. They do this by making the ganglion cells preferentially respond to spatial contrast in the input, and so eliminating responses to visual locations whose input is redundant with that of their neighbors. Consequently, the responses of retinal ganglion cells are much less correlated than those of the photoreceptors, making their representation much more efficient. One facet of this efficient encoding hypothesis is that the optimal receptive field transform should depend on the statistical properties, such as the correlation structure and intensity, of the input. This dependence has been used to explain adaptation, to changes in input statistics, of visual receptive field characteristics, such as the sizes of center-surround regions and the color tuning of retinal neurons, or the ocular dominance properties of striate cortical neurons [32], [34], [44], [45], [46], [47]. In the auditory system, information redundancy is also reduced along the auditory pathway [48]. Although this redundancy reduction was only investigated in the neural responses to sensory inputs rather than in the coding (STRF) transform leading to the neural responses, it suggested that coding efficiency is one of the goals of early auditory processes.

**Figure 2:**
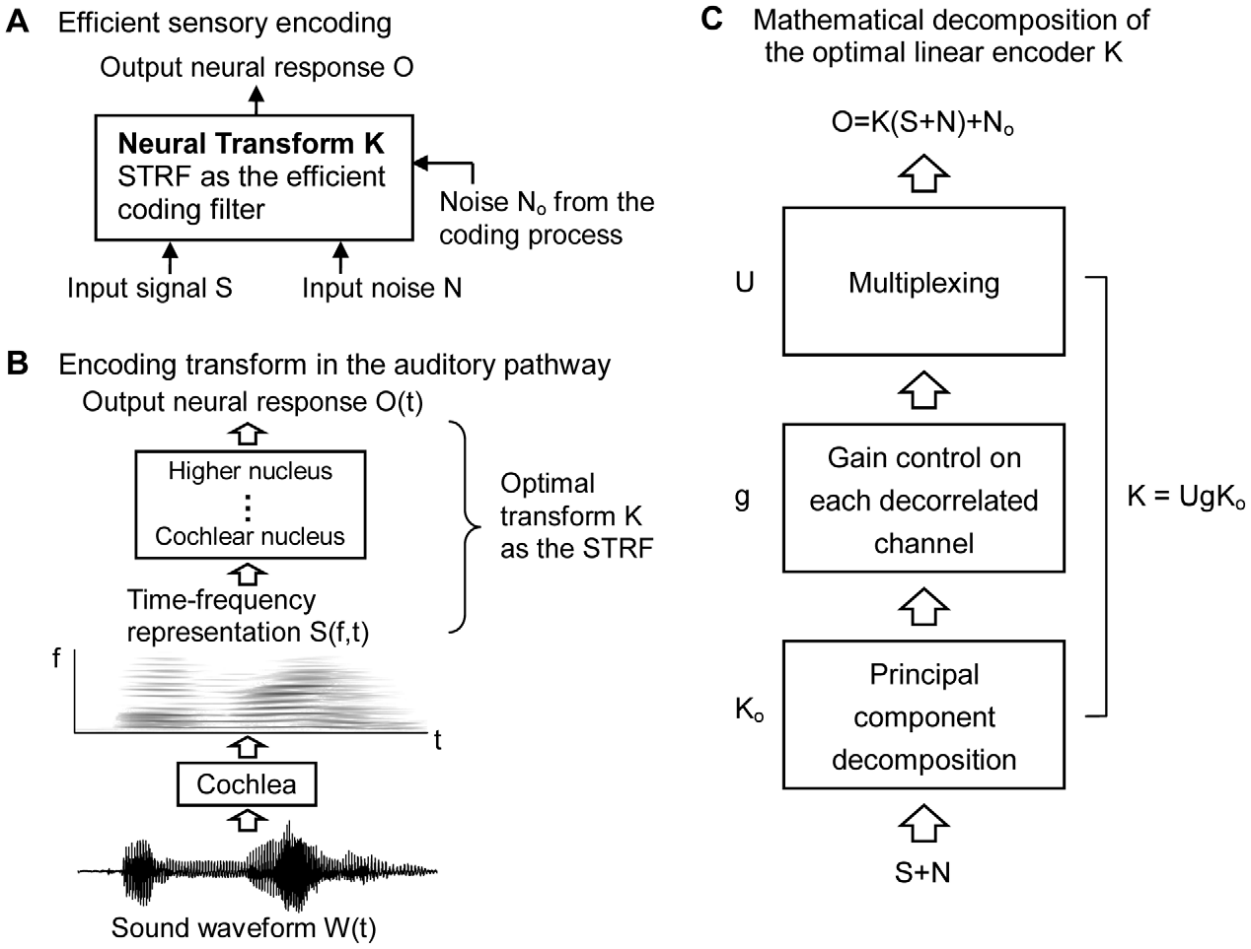
Formulation and components of efficient coding. (A) A schematic plot of the efficient encoding transform. (B) Signal transformation in the auditory system. The cochlea turns the time-varying waveform 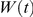 into a time-frequency representation 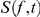, as the population activities of the auditory nerves, which is the input to the efficient encoding system. Signal and noise pass through a series of brain nuclei such as cochlear nucleus, superior olive, inferior colliculus, etc. The current work proposes that the effective transform STRF of the spectrogram that is collectively realized by these nuclei is, in its linear form, the optimal filter 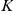 implied by the efficient coding principle. The output 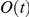 is the activity of neurons in a higher nucleus. (C) Three steps of signal flow within the linear encoding step 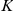 or STRF in (A) and (B). Note that these three steps are merely abstract algorithmic steps, rather than neural implementation processes for the effective transform 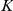 or STRF.

More formally, the efficient coding scheme is depicted in Figure 2A. The input contains sensory signal 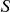 and noise 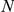 (e.g., input sampling noise). The net input 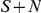 is encoded by a linear transfer function 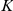 into output.
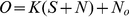
(2)which also contains additional noise 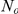 introduced in the encoding process. When the input has multiple channels, e.g., many different photoreceptors or hair cells, 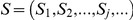 is a vector with many components, as indeed is 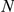. Output 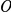 is a vector representing the neural population responses from many neurons. For output neuron 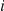, we have 
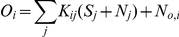
. Therefore 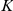 is a matrix, and its 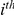 row 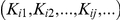 models the receptive field for output neuron 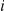 as the array of effective weights from input receptors 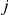 to output neuron 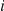. In the particular example when input neurons are photoreceptors and output neurons are retinal ganglion cells, 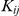 is the effective connection from photoreceptor 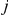 to ganglion cell 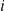 (implemented via the interneurons in the amacrine cell layers of the retina), and collectively, 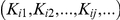 describe the linear receptive field of this ganglion cell. We consider the problem of finding an optimal 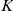 that maximizes the information extracted by 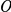 about 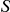, i.e., the mutual information 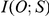 [49] between 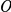 and 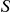 subject to a given cost of the neural encoding, which depends on the responses in a way we will describe shortly.

Therefore, the optimal 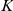 should minimize the objective function:
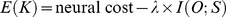
(3)where 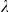 is a parameter whose value specifies a particular balance between the needs to minimize costs and to maximize extracted information. Neural costs can arise from various sources, such as the metabolic energy cost for generating neural activities or spikes [50] and the cost of thicker axons to transmit higher rates of neural firing. We follow a formulation that has been productive in vision [31], [34], and model the neural cost as
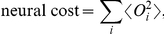
where 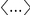 indicates the average over the stimulus ensemble. This gives
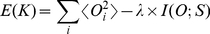
(4)It has been shown [29], [33], [51], [34] that the 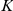 that provides the most efficient coding according to 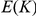 has the following properties. At high signal-to-noise ratio (SNR), 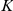 is such that 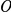 extracts the difference between correlated channels, and thus avoids transmitting redundant information. Hence, for example, in photopic conditions, retinal ganglion cells have center-surround spatial receptive fields which extract the spatial contrast of the input. By contrast, at low SNR, 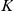 is a smoothing filter that averages out input noise instead of reducing redundancy. This avoids spending neural cost on transmitting noise. Hence, for example, in scotopic conditions, when SNR can be considered as being low, the receptive fields of retinal ganglion cells expand the sizes of their center regions and weaken their suppressive surrounds [52]. We will apply this framework to the auditory encoding to understand STRFs and their adaptation to stimulus ensembles.

## Methods

### Auditory encoding system and its comparison to vision

To apply the efficient coding principle to auditory STRFs, we borrow insights from vision by making an analogy between (aspects of) the auditory and visual systems. For simplicity, we start by ignoring input noise. While sound signals are typically air vibrations over time, at the input sampling stage, they are sampled as 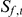 from a continuous time-frequency representation 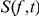, namely the response at time 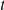 of a hair cell tuned to sound vibration frequency 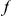. This is analogous to visual input sampling, in which the response of a photoreceptor at location 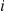 samples the light signal in the form of electromagnetic vibrations. Auditory hair cells are tonotopically arranged in the cochlea, so that neighboring hair cells are tuned to nearby sound frequencies. Therefore, at any instant 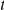 , the response pattern 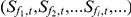 as a function of hair cell's location 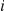 over the cochlea is an auditory “image” of the pattern of powers across sound frequencies, analogous to a retinal image. (In our formulation, we focus on sampling the intensity or power in 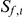, and ignore the phase of the sound wave at frequency 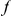. This is because (1) auditory nerve responses do not encode the phase except for low frequency inputs via phase locking, and (2), as mentioned, our goal is to understand the STRFs which do not concern the phase information.) While a retinal image is two dimensional in space (and one additional dimension in time), the auditory “image” at any instant 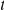 is one dimensional in sound frequency 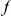. One may use time 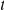 as the second dimension such that 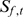 for all 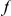 and 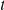 collectively can be seen as a single discrete sample of the two-dimensional auditory “image”. When input noise 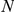 is included, input 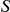 becomes 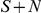.

As for vision, we explore whether the auditory STRFs can be partly understood by the goal of efficiently coding auditory information. The sensory input is sampled as 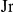, the responses of the cochlear hair cells. This input is encoded by the STRFs to give rise to outputs 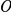 as the neural activities of a higher nucleus, such as the inferior colliculus (IC) or the auditory cortex (Figure 2B). The STRF is then analogous to a spatial receptive field, such as that of the retinal ganglion cells. Thus the STRF should be determined by the statistics of the auditory inputs, and in particular, the correlation 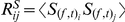 between different inputs 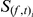 and 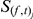, where 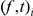 labels a particular spectro-temporal combination of a frequency value 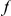 and time 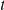. Note that for 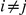, the frequency 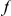 or 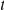, but not both, in the two indices 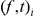 and 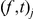 may be equal. (Here, for simplicity we assume, or pre-process the signal, such that all inputs have zero mean, i.e., 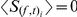, just like the input signal fluctuation 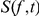 around the ensemble average in the definition of the STRF in equation (1)). As in vision, natural auditory inputs express substantial correlations between inputs of neighboring frequencies and at neighboring temporal instances. When the input SNR is sufficiently high, an optimal STRF should reduce these correlations to achieve efficient transmission. Such an STRF will have neighboring excitatory and inhibitory regions in the frequency-latency domain, making the neuron be tuned to spectro-temporal contrast and be insensitive to the spectro-temporal redundancy.

### Auditory STRF filter as an efficient coding transform

The general formulation and derivation of the efficient coding transform 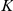 (or STRF) can be found in its application to vision [34]. Here we outline these results and illustrate their consequences for auditory coding. Let 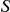 be the input with 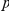 input channels:
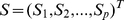
(5)(superscript T denotes vector or matrix transpose). These 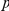 input channels may correspond to 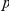 auditory nerves if we omit the temporal dimension, 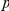 time instances if we focus on a single frequency channel, or they may correspond to 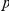 spectro-temporal labels 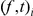 for 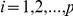. Let the input correlation be described by correlation matrix 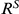 with elements 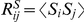. The optimal transform 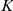 that minimizes  in equation (4) can be decomposed in three steps (Figure 2C): (1) a principal component transform to de-correlate the inputs, (2) gain control of each principal component, (3) an ortho-normal or unitary transform on the array of the gain-controlled components to arrive at various output channels. We now elaborate and elucidate these three steps.

The first step is a coordinate rotation, or ortho-normal transform, 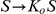, by an ortho-normal matrix 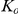 that de-correlates the input channels such that each of the channels in the transformed signal 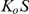 contains a principal component of the original signal. We denote these principal components as 
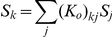
, with sub-index 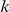 (instead of 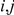) as the indices of the de-correlated channels (later, we also use 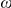 to denote the de-correlated channels in the temporal domain, or 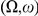 in spectro-temporal domain). Since the correlation between 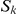 and 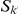 is 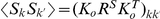, decorrelation between principal components implies that 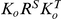 is a diagonal matrix, with 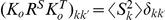, where 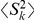 is the 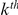 eigenvalue of matrix 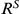 and also the average signal power of the 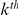 principal component 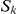. As we will see later, when the input correlation 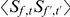 depends mainly on the differences 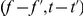 in frequency and time, it turns out that 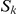 (with the index 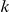 denoting the spectro-temporal modulation frequency 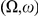) is the amplitude of a dynamic or moving ripple that some experiments use to estimate the STRFs of cortical and midbrain neurons [17], [18], [19], [16], [2].

The second step is gain control 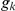 on each component 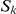, giving output 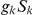. Including noise 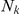, which is the original input noise 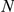 projected to the 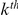 channel by the transform 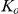, and the encoding noise 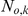 (in the decorrelated 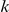 space), the total output becomes 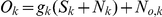. It can be shown (see [34]) that the gain 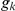 that minimizes 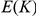 in equation (4) is determined by the input signal-to-noise ratio 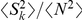 to satisfy
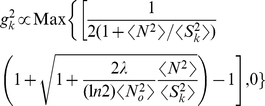
(6)where 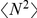 is the variance of 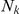, and also of the input noise 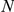 (assumed to be independent, identically distributed and Gaussian in each channel) , and 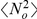 is the variance of the encoding noise 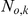 in each channel 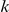 (and of the encoding noise 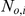 in each 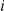 since different encoding noise channels are also assumed to be independently and identically distributed).

Note that the total noise at output neuron 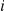 is 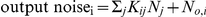. One effect of the encoding transform 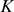 is that noise corrupting different output neurons can be correlated, even when the original input noise is independent. The additional encoding noise 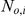 could also be correlated in different output neurons, since it could also reflect a common origin in intermediate stages of the encoding processes. Our assumption of independence between 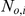 and 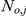 for 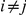 is thus a simplification for mathematical convenience.

Since all the variables are assumed to be Gaussian, each output 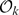 extracts the following amount of information
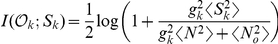
about the input 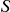 and has an output power 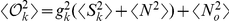. Since different output channels 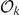 from different 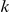 are decorrelated from each other, the quantity 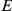 in equation (4) is
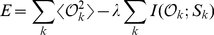
(7)One can then verify that 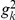 in equation (6) indeed minimizes this 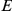 since 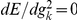 at that value. Note that if 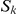 is the amplitude of a moving ripple indexed by 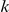, 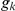 will be the sensitivity of the neuron to the moving ripple.

We can write these two steps as the product 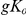, where 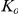 is the principal component transform, and 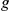 performs the gain control. 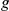 is a diagonal matrix with diagonal elements 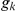. The net output is then 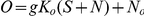. Consider imposing on this transform an orthonormal or unitary transform 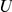 (with 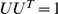), the third step in building the efficient coding filter 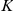, giving 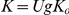. It follows [34] from the properties of unitary matrices that neither the first term nor the second term in 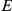 in equation (4) will be affected by 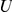 (at least when signal and noise are Gaussian and when the components of 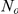 are independent and identically distributed).

Each row vector of the matrix 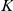 determines the receptive field of a particular output channel or neuron. Without 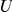, 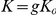 would specify receptive fields that would be gain controlled eigenvectors or principal components of the input correlation matrix. For example, they would look like ripples covering the entire spectro-temporal range. An appropriate choice of non-trivial 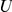 will alter the receptive field shape dramatically, giving rise to receptive field properties found in real neurons such as a finite span in input channel space. For example, if we consider only the input frequency channels 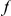 for auditory inputs and omit the time dimension, we may prefer that the STRF for an output neuron to be selective to only a finite band of input frequencies such that the neural responses 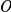 resemble periphery inputs 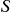 while maintaining coding efficiency. It can be shown [34], [35] that this can be achieved by choosing 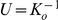, such that 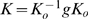. We will use this choice, 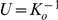, in building our STRF in frequency domain. However, insensitive to the exact form of 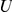, the critical feature of the STRF comes from the gain 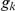 specified in the second step of the encoding model (as long as one does not impose additional computational goals that may restrict the final STRFs, see Discussion). We will show later that 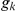 often corresponds to the modulation transfer functions (MTFs, also called ripple transfer function, RTF,in different literatures) of the STRFs.

We now apply this general framework to the case of auditory encoding. Sound spectrogram 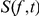 is derived from the sound waveform 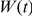 as follows. The first step is to perform a temporally-windowed Fourier transform of 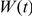 to obtain the sound spectrum 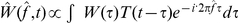 as a function of time, where 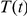 is a temporal window function (e.g., 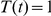 for 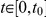, 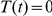 otherwise). Since the cochlea performs approximately a log scale frequency analysis, we first let 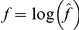 to obtain 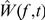 (although the more accurate form would be 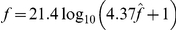 [53]). Then the input power in 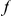 is 
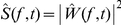
. One may employ a further logarithmic transform 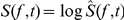 to characterize the cochlear response better (through capturing the compressive input/output transform realized by processes in the basilar membrane and hair cells) [54], [55]. However, this further logarithmic transform is not essential for our formulation, and, as pointed out previously [56], it does not significantly affect the qualitative characteristics of the empirical STRFs. If one omits this logarithmic transform, then 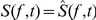. We then subtract the mean 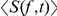 from 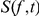, and, for simplicity, denote the resulting zero mean signal still by 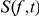, as in the definition of STRF. We next consider discrete samples 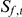 of the continuous 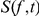. This leads to the input correlation matrix 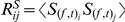.

Finally, we follow the three encoding steps above to obtain the optimal encoding transform as 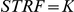. In the sub-section “The spectral filter SRF”, we discuss the simple case in which the temporal dimension 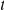 is omitted. Then, the input vector (equation (5)) is 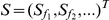, and the input correlation matrix is 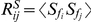. The efficient encoding procedure specifies the optimal spectral receptive field (SRF) 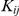 for neuron 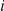, with 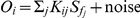. When the temporal dimension is included 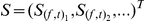, 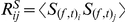, and efficient coding specifies the optimal STRF as input weights or selectivity associated with the spectrogram 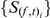.

It is apparent that the optimal SRF and STRF depend on input statistics via the input correlation 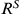 and the input SNR (through the steps 1 and 2 in the encoding scheme). Therefore, when the stimulus ensemble changes, altering the input correlations and signal intensity, the form of the encoding receptive field should adapt in order to maintain encoding optimality. We propose that it is this that explains the input ensemble dependence of the STRFs.

A special class of input statistics has translation invariant correlations, i.e., with 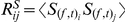 depending only on the differences 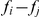 (quantified in octaves) and 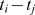. This is a reasonable approximation of the input correlations in natural auditory scenes under two conditions. The first is that a local frequency range is considered that is not much larger than the range of the frequencies to which a neuron is sensitive, i.e., in the perspective of a neuron, the dependence of 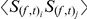 on the frequency is mainly through 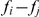. This is analogous to approximating spatial correlation of visual inputs as translation invariant to understand the retinal ganglion cell's spatial receptive fields although the spatial sampling density varies substantially with input eccentricity [31], [34]. The second is that the environment is statistically stationary, as then the correlations in time depend only on the temporal difference 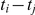. It can then be shown that [34] the principal components are 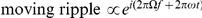, each of which has a 2D modulation frequency 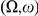, which can be indexed by 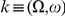. The first encoding step is then a 2D Fourier transform 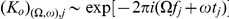 of the input 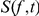 to obtain 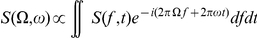. Meanwhile, the original input can be written as 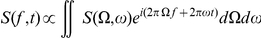, i.e., as a weighted sum of the moving ripples [19]. The second encoding step determines the gains for the ripple amplitudes 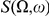 [34] as
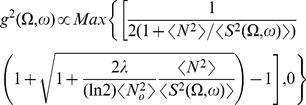
(8)i.e., replacing 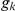 and 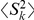 in equation (6) by the corresponding 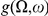 and 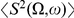. If 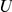 is chosen as the inverse Fourier transform
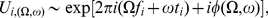
(9)with an extra phase function 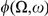, then the encoding transform is 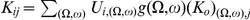. This gives
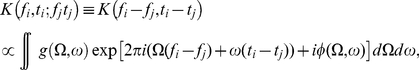
(10)which depends only on the differences 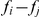 and 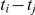. Applying this transform to input 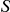 to give output 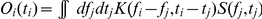, we see, by comparison with equation (1), that the STRF is 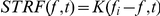. This is a temporal filter tuned to sound frequency with a tuning pattern governed by 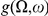, and centered around frequency 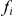. Changing the center frequency from 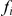 to 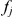 is like shifting from one output neuron 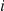 to another neuron 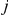. Altering the phase 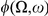 in equation (9) alters the STRF shape, in particular to ensure its temporal causality. In physiology, modulation tuning function (MTF) is often mentioned as the Fourier transform of auditory receptive field [19]. Therefore, it is clear from equation (10) that the gain profile 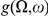, which is determined by efficient coding, corresponds to the magnitude of the MTF. However, the shape of an STRF is determined by the phase as well as the magnitude of the MTF, and efficient coding does not strongly constrain the phase. Therefore, while we will illustrate the general properties of some example STRFs predicted by the theory by choosing particular 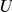 transforms (governed by the additional requirements of spectro-temporal locality and causality), in the Results, we will generally compare physiological data to the magnitudes of the MTFs that the theory predicts.

In the Results, we will discuss the efficient coding framework for situations both with (e.g., to study temporal aspects of STRFs) and without (e.g., to study their spectral aspects) translation invariance in input statistics.

## Results

To illustrate how the framework explains and predicts physiological experiments, we first discuss a few examples when the temporal or the spectral dimension is omitted, and then show a full spectro-temporal STRF.

### The spectral filter SRF

**Figure 3:**
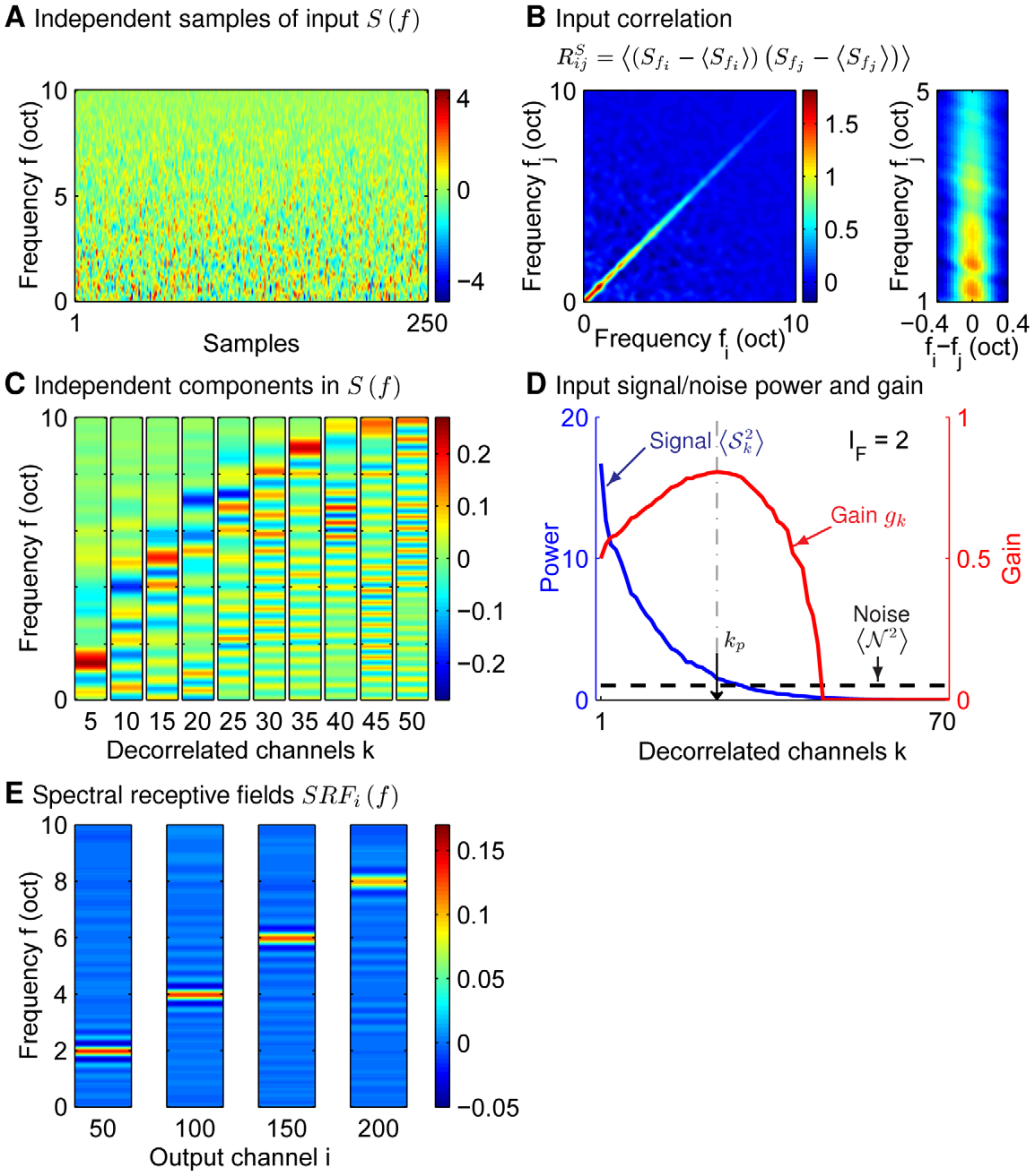
Simulation of the efficient spectral kernel SRF, when the temporal dimension is omitted. (A) 250 samples of input spectra 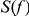, each of which is smoothed Gaussian white noise in the frequency domain (equations (11–13), 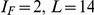). (B) Correlation between different frequency channels 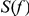. Left: Correlation 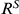; Right: an zoomed-in view, as 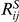 vs 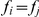. (C) Ten examples of eigenvectors 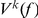 of the correlation matrix 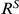 in B; each is an independent component in 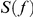. Smaller indices 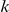 are associated with larger eigenvalues. (D) Gain profile (peaking at 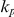), and signal and noise power in decorrelated channels. (E) Four examples (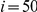, 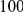, 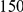, and 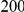) of spectral receptive fields 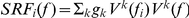; each prefers input frequencies around 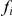.

We first omit time, treating the input 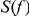 as varying only in frequency. In this case, the encoding filter reduces from being an STRF to an SRF. We take 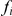 as one of 250 discrete values 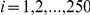, from low to high frequencies; hence input 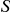 is a one dimensional vector 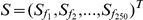. In simulations, input sample 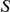 is generated by smoothing a random noise vector 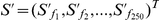 (Figure 3A), with all the components 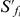 taken to be independent, zero mean, unit variance, Gaussian noise. Specifically
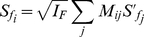
(11)where 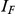 is a factor to scale the overall input power intensity, and 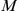 is the smoothing matrix with elements
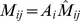
(12)explained in detail below. Here  controls the scale of the signal 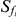, which decays with 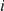 (like in an environment in which high frequency sounds do not propagate well), and 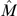 is a normalized smoothing matrix with elements 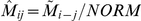, in which
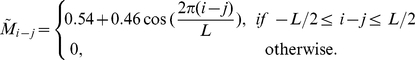
(13)

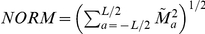
 is a normalization constant, and 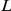 controls the range of frequency difference 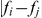 for significant correlation coefficient between the variation of 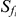 and that of 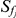.

Consequently, each 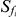 is also a zero mean Gaussian random variable, and the input correlations comprise a 250×250 matrix 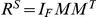. One could also estimate 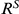 from input samples 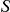 (as when animals adapt their auditory system to environmental sound through experience), in which case element 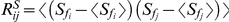. Figure 3B illustrates 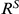 (obtained numerically from 250 samples of 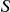 in Figure 3A, of course one could use more than 250 samples to estimate 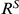) for 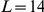. The correlation 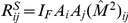 scales with strengths of the original signals 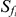 and 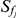 through the scales 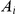 and 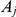, and so decays with frequency 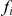 and 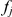. Thus the statistics of the stimulus ensemble are not translation invariant in the spectral frequency 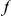. Nevertheless, the correlation coefficient
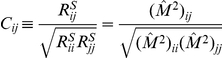
does depend mainly on the (frequency) difference 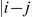, since 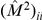 is almost independent of 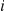 and 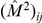 depends mainly on 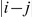 except for the very small or very large 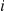 and 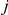. This is evident in the fact that the rate of decay of 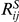 with the difference 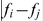 in Figure 3B is almost constant. Since the stimulus ensemble is not translation invariant, we will use the general formulation to obtain the SRF. From 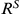, we obtain its 250 eigenvalues and the corresponding eigenvectors. Each of these is a vector with 250 components. We list them in the order of descending eigenvalues, denoting the 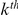 eigenvector as 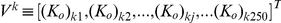, and placing it as the 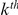 row vector of the 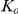 transform matrix. Figure 3C depicts the eigenvectors for 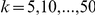, where smaller 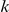 is associated with a larger eigenvalue. Each principal component or eigenvector can be seen as a special input spectrum pattern 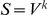, while a general input 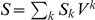 is a linear sum of the principal components with weights 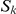. The first encoding step is thus a transformation of the original input 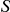 by 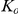 to obtain the decorrelated signal 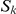, for 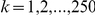. The average power in 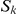 is the 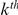 eigenvalue of matrix 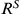


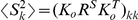
The eigenvectors look roughly like oscillating waveforms (spectral oscillations) with different oscillation rates, and are comparable to the sinusoidal bases in the Fourier transform. They also resemble the “ripples” used in physiological experiments. This is because the input correlations are roughly translation invariant, at least within a small range of frequencies in which the signal power 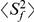 is roughly independent of 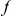 (just like in vision when the statistics of inputs sampled at the retina can be seen as roughly translation invariant within a local region). Also note that smaller or larger 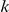 is associated with eigenvectors with fewer or more oscillations. This makes 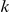 relate monotonically to the spectral modulation frequency (corresponding to the “ripple frequency” 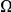 in physiological experiments). Larger eigenvalues, i.e., larger signal powers 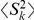, are associated with fewer spectral modulations or smaller indices 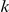, because inputs of more similar sound frequencies are more correlated with each other, i.e., 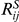 decreases with increasing 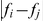. The analogy between the eigenvectors and the Fourier bases can be understood as follows: if 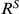 is strictly translation invariant, then the eigenvectors are sine waves with different spectral modulation frequencies 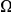. The eigenvalues are the Fourier transforms of 
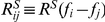
, and hence they decrease with the modulation frequency 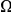 because 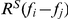 is non-negative and decreases with increasing 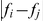.

The second encoding step is to assign the gain 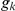 to each of these channels 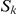 according to equation (6), giving 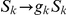 (see Figure 3D; 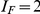, 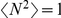 and 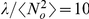). Note that while the signal power 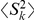 decreases with increasing 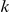, the gain magnitude 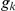 first increases with 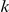 and then decreases and drops to zero at higher 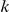.

The gain for small 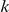 is low since the SNR 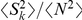 is high enough to make amplifying 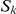 less necessary. From equation (6) [34],
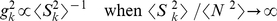
(14)This implies that 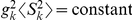 for sufficiently large SNRs. When each principal component 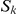 is a modulation frequency mode, this gain profile 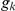 is often called whitening. At smaller signal powers, the gain increases so as to utilize the channel's dynamic range fully. However, when SNR is too small, for example, when noise power is higher than signal power 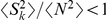, gain decreases with decreasing 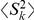 [34]. This is because such input components are dominated by noise, and amplifying noise increases neural cost. Thus, in general, when 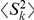 decreases with increasing 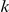, the gain profile has a band-pass shape, first increasing, and then decreasing with increasing 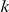 (see the red curve in Figure 3D). The peak of the gain occurs at 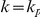, where 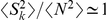.

Third, taking 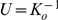 in order to localize the receptive Fields as best as possible, the overall encoding transform is 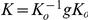. Here, the gain matrix is diagonal having elements 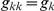. When 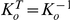 (as when the eigenvectors are real and othornormalized)
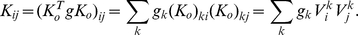
As the overall encoding transform gives outputs 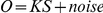, where 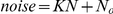, the 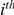 output neuron 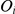 has its SRF as a vector of weights for inputs 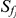 of various frequencies 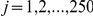


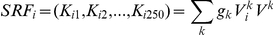
It can thus be seen as a weighted sum of the eigenvectors 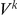 of the input correlation matrix, with weights 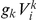 for output neuron 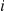. Figure 3E shows SRFs for four different output neurons (or channels 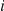). These SRFs have different preferred frequencies 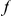, so that the preferred frequencies of all the output neurons span the whole input frequency range. The shapes of the SRF depend on the input statistics via the dependence of 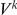 and 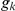 on the input correlation matrix 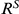. In particular, for sufficiently high input SNR, while a neuron is excited by its preferred frequency, it is suppressed by nearby frequencies. This form of contrast enhancement achieves a measure of decorrelation between neighboring output neurons that would otherwise reflect the strong correlations between neighboring frequencies. For SRFs tuned to higher frequencies, the center excitatory regions are larger and the surround suppression is weaker. This is because SNRs are weaker for higher frequency inputs (the dependency of SRF on SNR will be discussed in the next sub-section). If the input statistics are strictly translation invariant, the SRFs for different output channels will have the same shape, and will just be centered on different frequencies.

### Adaptation of SRF to input signal-to-noise ratio

When sound intensity decreases, the basilar membrane in the cochlea undergoes a smaller vibration. This decreases the magnitudes of input signals 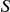, and so, if the level of the noise stays unchanged, the signal-to-noise ratio 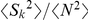 will decrease. This will change the optimal encoding gain 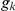 via equation (6), and thus change the final SRFs. In our example, we simulate the change in input intensity by changing 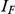 in equation (11).

**Figure 4:**
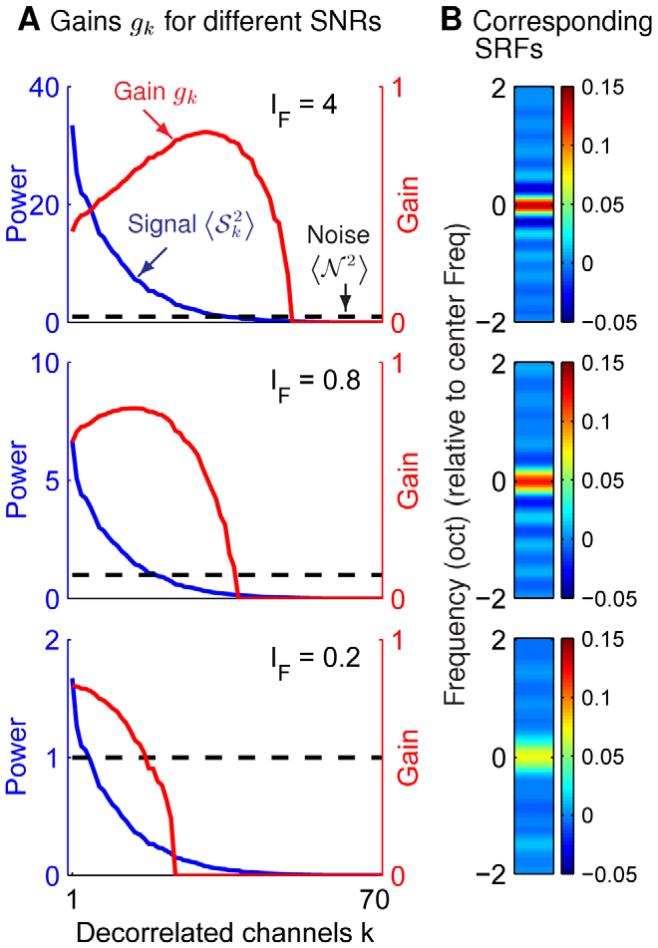
The effect of signal-to-noise ratio (SNR) on gain 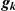 and the spectral receptive field (SRF). Same stimulus ensemble as in Figure 3A except the overall SNR has been scaled by 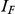. (A) Gain control (red), signal (blue), and noise power (black) under high, medium and low SNR. (B) The corresponding SRFs of one output neuron (channel #120) in the three SNR cases.

Figure 4A shows three example input intensity profiles 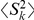, and the corresponding gain profiles 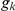. While an overall change of input intensity merely scales the profile 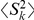 up and down, the gain profile 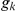 does not trivially scale up and down. When input intensity decreases, the 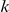 at which 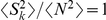 becomes smaller, thereby decreasing the 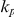 at which 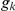 peaks. Consequently, the gain profile turns from being band-pass to being low-pass (Figure 4A).

The non-zero gain at higher 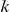 implies sensitivity to weaker principal components with more spectral oscillations (or higher “ripple frequencies”). Thus, as input intensity decreases, the overall SRF filter changes in two ways (Figure 4B): (1) it fluctuates less (i.e., has fewer excitatory and inhibitory regions, and with decreased strength inhibitory regions); (2) the width of the excitatory and inhibitory regions increases, as the result of losing contributions from spectral modulations 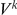 with higher modulation frequencies.

The insights from Figure 4B can help to understand the difference between the four SRFs in Figure 3E. Given the 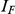 as in Figure 3, one may divide the whole sound frequency range into two ranges of equal bandwidth, one for the lower and the other for the higher 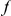
's, and treat the two ranges as if they were two different stimulus ensembles. If one ignores the overall sound frequency difference between these two ensembles, then these two ensembles differ from each other only in their SNRs, with a higher SNR for the ensemble for the lower sound frequencies 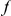. In this perspective, one can understand why a SRF tuned to the lower frequencies in Figure 3E has a narrower excitatory region and a stronger surround suppression than a SRF tuned to higher frequencies, using the insights gained from Figure 4. (In comparing Figure 4B with Figure 3E, one should note that each SRF in Figure 4B is depicted by zooming to the frequency region around the preferred frequency 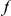 of the SRF.) One may even view the four SRFs in Figure 3E as if they were each exposed to one of the four different stimulus ensembles that differ in SNRs (and in sound frequency 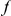, and we ignore this difference). Within each of these stimulus ensembles, the input statistics may be seen as approximately translation invariant, since 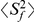 is almost independent of 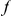 and the correlation 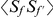 is approximately only a function of the frequency difference 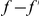 within a small range of frequency 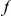.

### Adaptation of SRF to input signal correlation

**Figure 5:**
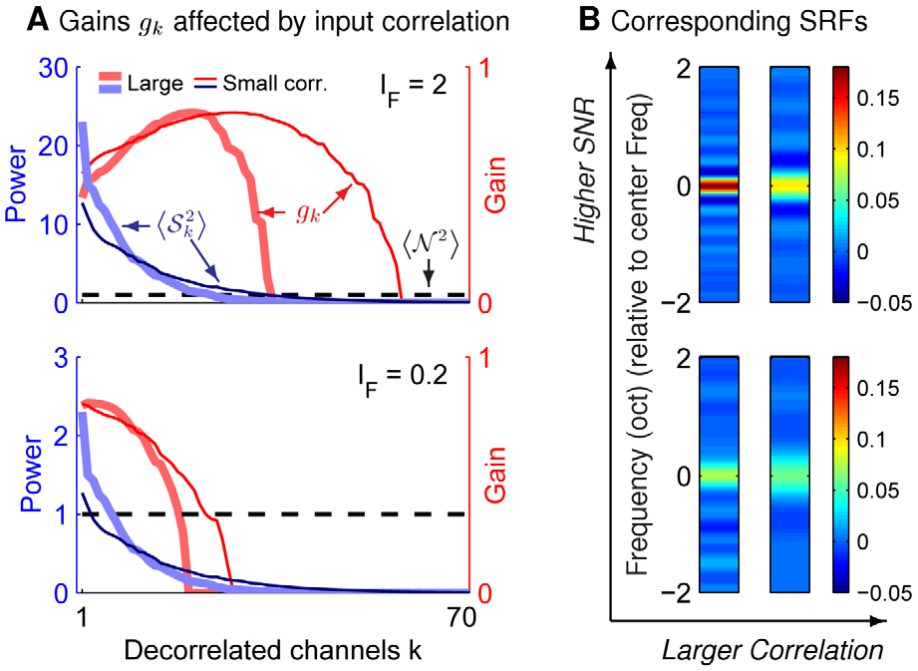
Adaptation of gain 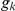 and spectral filter kernel SRF to input correlations under high/low SNR. Same input ensemble as that in Figure 3A, except that the smoothing parameter, 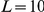 and 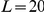, are set for short and long range correlations, respectively. Analogous figure format as in Figure 4, with added illustrations of the adaptation to input correlations. The thick and thin curves correspond to quantities for inputs with large and small correlations respectively, blue/red curves plot signal power 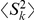 and gain 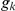 respectively.

As well as adapting to the input SNR, the SRF can adapt to the signal correlations in the input. These can also vary across auditory environments. We generate two stimulus ensembles (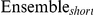 and 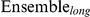) based on equation (11), with short and long range (in frequency space) correlations between inputs 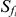 and 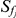 of different sound frequencies. We do this by setting the smoothing length 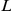 in equation (13) to be 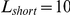 and 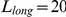. Since short and long range correlations give respectively smaller and larger correlations or degrees of input redundancy, in this paper, we use the terms short/long-range and small/large correlations interchangeably. The two stimulus ensembles are made to have the same overall signal power 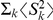, and consequently their 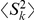 vs. 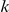 curves cross each other at a particular frequency 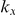 (Figure 5A). In 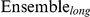, signal power 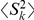 is more concentrated in lower 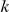
's, and the “bandwidth” of gain, i.e., the range of 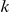
's with substantial 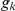, is consequently narrower.

If 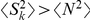 at 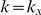, the 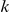 at which signal power 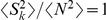 is larger in 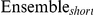 (Figure 5A, upper panel, 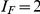, 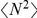 = 1, 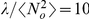). Thus, the frequency 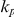 at which gain 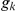 peaks is also larger in 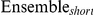. If the SNR is lower, so that 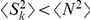 at 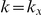, then 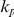 is instead smaller in 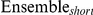 than in 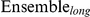. However, this is less apparent since gain profiles in both ensembles become “low-pass” in 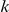 implying that there is no obvious “peak position” (Figure 5A, lower panel, 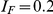 ). Nevertheless, the cutoff frequency 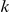 where 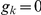 is always smaller for 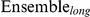 (Figure 5A), and the optimal SRFs for it consequently enjoy a greater spectral extent (i.e., the SRFs are non-zero for a larger range of 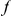 (Figure 5B). Intuition for this effect is that for it to be effective as either a contrast enhancing filter at a high SNR, or a smoothing filter at a low SNR, the SRF's spectral extent should match the range of the input correlations.

### The temporal filter TRF

We can similarly ignore the frequency dimension of the input to understand the temporal receptive field (TRF). This is determined from the way 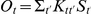
+noise, the input temporal sequence 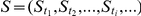 is transformed to the output temporal sequence 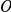. In a statistically stable auditory environment, the input correlation should be time shift invariant, i.e., 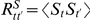 should depend only on 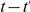. Denote 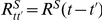. Then, the de-correlating transform 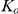 should just be a Fourier transform 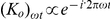 with the principal component 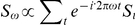 being the Fourier Amplitude of the relevant mode. Here we use index 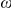 instead of k to denote the principal component to signify the association with the temporal Fourier amplitude. The average power 
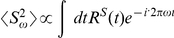
 is simply the Fourier transform of the input temporal correlation. If we set 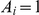 in equation (12) to generate inputs with shift invariant correlation, then 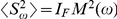 where 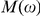 is the Fourier amplitude of 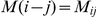. The gain control 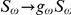 in the second encoding step is determined by equation (6) (substituting 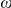 for 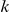). The final TRF will be the transform 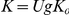 given an appropriate choice of 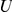.

**Figure 6:**
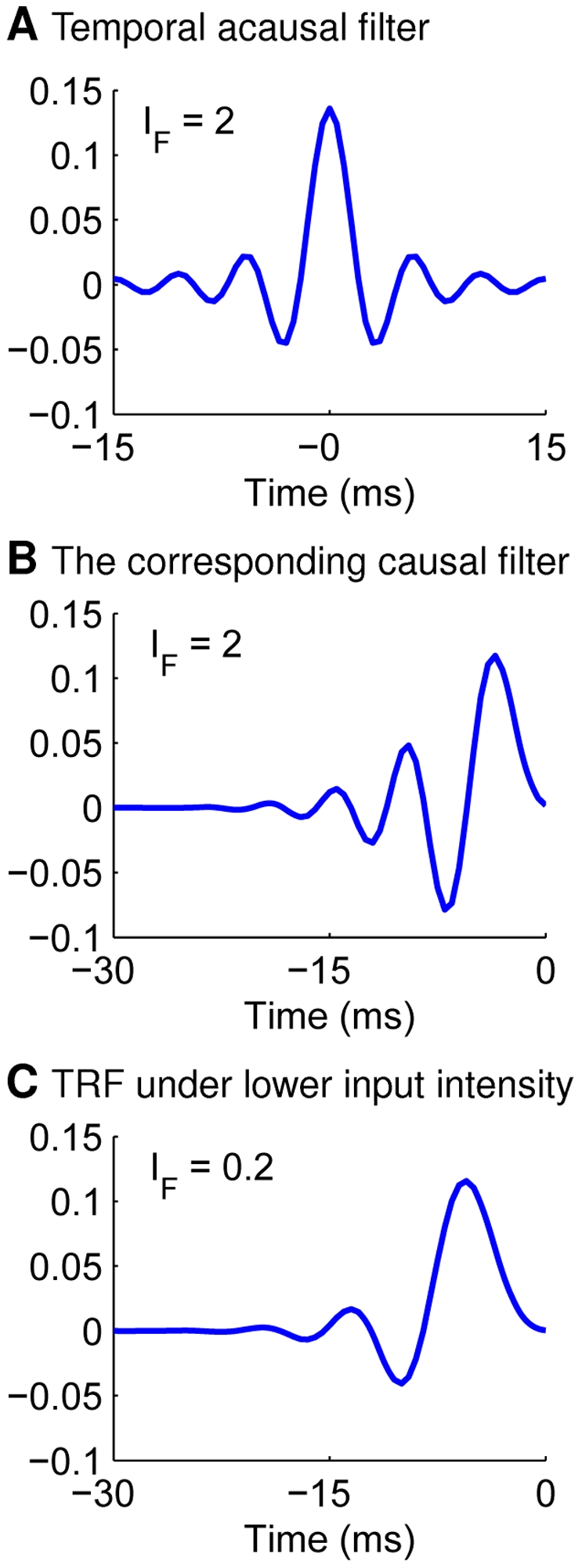
Simulation of temporal receptive field TRF, when the spectral dimension is omitted. The same stimulus ensemble is used as in Figure 3A, except the factor 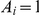 in equation (12) to ensure translation invariance of correlation. (A;B) Demonstration of transforming an acausal temporal filter (A) to its causal minimum-phase counterpart (B) at a relatively high input SNR. (C) TRF for a relatively low input SNR.

However, the actual procedure to obtain the TRF is trickier in that the 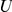 transform in the third encoding step to give the overall 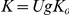 has to be chosen to satisfy the causality constraint. That is, the output 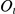 at time 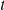 should only depend on past input 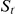 for 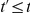, i.e., 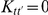 for 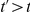. Moreover, it is better for the TRF to have a short temporal span and latency, an outcome that can be achieved by assuming that the optimal temporal filter 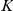 has a minimum phase-shift [57]. Short latency can feasibly be implemented by neural synaptic and membrane mechanisms that typically have time constants no longer than a few hundred milliseconds [58]. Hence, these offer credible constraints on the TRF. Note that if we choose 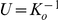, i.e., 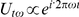, then 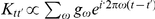
would be an even function of 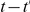 and thus not a causal temporal filter given gains 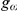 that are all real. The filter 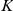 can be made causal and minimal phase by choosing another 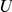 simply as 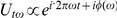 with a particular phase function 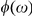, so that 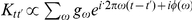. Instead of directly obtaining this phase function 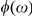, we can also equivalently obtain this minimum phase shift causal filter by transforming the acausal 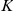 using standard procedures in signal processing theory as follows (see [57] for the proof). Given a non-causal filter 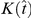 with finite non-zero values in discrete time 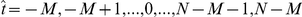, first let 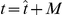 to make a causal filter 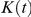 whose nonzero values are at 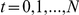. Second define
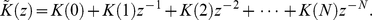
Among the 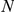 complex roots of the equation 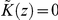, let 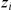 denote the roots with 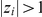 and 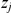 the other roots with 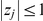. Third, let
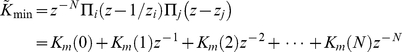
The coefficients 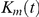, 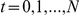 are the values of the desired causal minimum phase filter. One example of this process is demonstrated in Figure 6A (before the minimum phase adjustment) and Figure 6B (after the minimum phase adjustment)(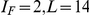).

The temporal kernel also depends on the SNR and the input correlations. The change in 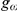 when sound intensity becomes lower is similar to that in the spectral case: from band-pass to low-pass. A temporal kernel under lower SNR is demonstrated in Figure 6C. The changes in 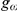 and TRF with input correlations are analogous to those in the spectral case as well (figure not shown).

### The two dimensional STRF

Finally, we show examples of the two dimensional 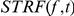. Here, we extended the assumption of shift invariance in the input correlations to the spectral dimension for the convenience of calculation. This assumption is reasonable when individual STRFs cover sufficiently small ranges of frequencies that the correlation in the spectral space is almost translation invariant within that range, as we see in our SRF examples. Then, spectral and temporal dimensions can be de-correlated at the same time by performing a 2-D Fourier transform on inputs 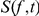, with the moving ripples as decorrelated channels, each denoted by a 2D index 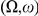 marking the spectral and temporal modulation frequencies.

**Figure 7:**
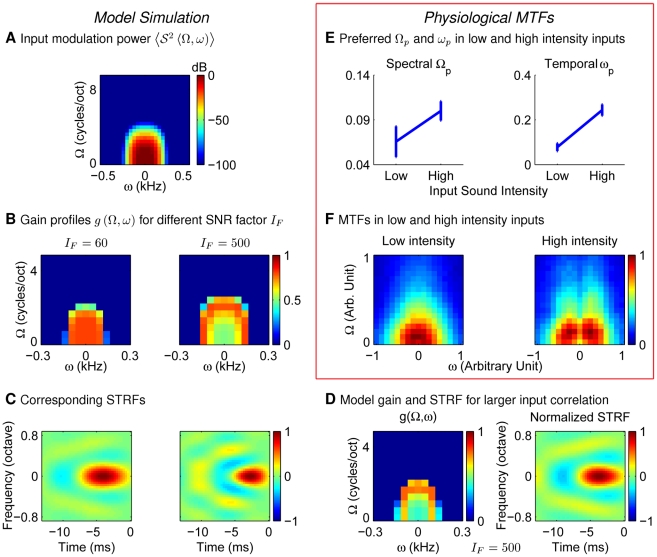
The 2D STRFs/MTFs implied by efficient coding and found physiologically. (A) input power 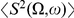 (equation (15), 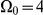, 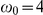) in decorrelated channels. (B, C) MTF profile 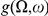 and the corresponding STRFs with two SNRs (scaled by 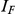
's). (D) 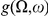 and STRF as in B;C (when 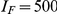) except with larger input correlations (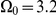, 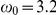 in equation (15)). (E;F) Modulation transfer functions (MTFs) and their properties at low and high input sound intensities averaged over 40 IC neurons from Lesica and Grothe [7]. Here, 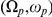 is the spectral-temporal modulation frequency where the MTF peaks. Modulation frequencies in E and F are normalized by the same value across cells and intensities. Error bars in E indicate standard errors. The magnitude patterns of the MTFs for all neurons are normalized to peak value 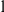. Their average across neurons at each input intensity is then normalized to the same peak value and displayed in F.

Let the signal power in the de-correlated channels 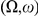 for input 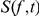 be 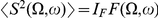. Here, 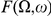 typically decays with modulation frequency 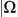 and 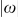 since most natural inputs have input correlation 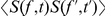 that decays with 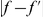 and 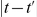. 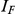 is a scale factor that controls the SNR. We use the following example in our simulations
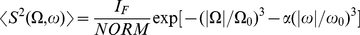
(15)where 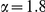, 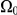 and 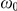 are parameters that control input correlation, and 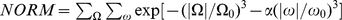 is a normalization factor. Figure 7A shows an example with 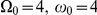 According to equation (8), the gain 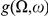 can be obtained as shown in Figure 7B (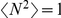, 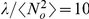, and 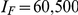). In particular, in the frequency range 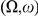 in which noise is negligible relative to the signal, the gain
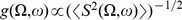
(16)specifies the whitening filter of equation (14). This gain profile changes from being a band-pass to a low-pass two dimensional filter as the SNR is lowered.

As we noted before, efficient coding predicts the gain 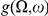, or the modulation transfer function (MTF), but does not precisely determine the STRF shape. The latter depends on the less constrained 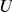 transform. Therefore, we qualitatively compare our 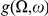 for two different 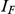
's with the MTFs obtained from physiological experiments under two different input sound levels. Figure 7E and Figure 7F are obtained from data on STRFs of 40 cells in the inferior colliculus of animals exposed to natural rain sound at low and high sound levels [7]. We first did a two-dimensional Fourier transform on the STRF of each cell to obtain its MTF. Then the spectral modulation frequency 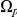 and the temporal modulation frequency 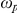 where the MTF has its maximum value were identified and normalized by a fixed value across cells. The average 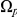 and 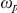 across all cells are shown in Figure 7E. These two “peak frequencies” both increased when sound intensity increased. The physiological MTF averaged across all cells (Figure 7F) also becomes higher pass, both spectrally and temporally, under higher sound intensities, as predicted by efficient coding (Figure 7B).

For completeness, we illustrate in Figure 7C the model STRFs from the gain profiles 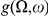, using an inverse Fourier transform with a proper phase function 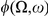 as the candidate 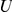 matrix. Specifically, the model STRF is
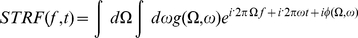
where the phase 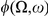 is chosen to make the STRF causal, and with minimum phase shifts in the temporal dimension. In practice, the STRF is obtained as follows, by extending our method for obtaining the causal 1-D TRF. For each 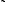, we first obtain the temporal acausal filter
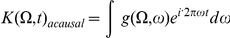
and then transformed this into a causal minimum phase filter 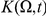 as for the one dimensional TRF filter. The final two-dimensional STRF is then
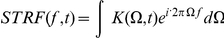
In general the model STRF has its highest amplitude at the preferred frequency on the spectral axis and for short latencies (i.e., the early part of the temporal axis). At low 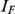, the STRF has a large excitatory region and a weak inhibitory surround (Figure 7C). At larger 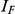, the STRF involves more excitatory and inhibitory regions with an increased inhibitory strength. Overall this has a more band-pass gain profile. Meanwhile, the bandwidth for the gain 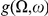 increases with 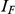, thus shrinking the width of the main excitatory region. Therefore, adaptation to higher sound levels makes the frequency-time tuning curve sharper, or equivalently more narrowly tuned and so, at a single cell level, supporting a more precise read out of the time and frequency of auditory input. Qualitatively, physiologically observed STRFs adapt to the input intensity in the same way [7] (also see [14]).

The model also predicts changes to MTFs and STRFs for different input correlations. Figure 7D shows the gain function 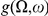 and STRF for an example in which the input has longer-range correlations in both spectral and temporal dimensions (we set 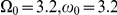 while holding 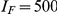 as in the high SNR case in Figure 7B and 7C). The peak modulation frequency in 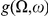 is decreased, and the excitatory region is wider compared with counterparts in Figure 7B and 7C at high SNR. This is consistent with our 1-D results in the spectral dimension (Figure 5).

## Discussion

### Summary of findings and predictions

In summary, this study set out to understand the computational role of auditory spectro-temporal receptive fields (STRFs). In particular, we generalized previous work [26] by proposing that STRFs are efficient codes for inputs which retain maximal information for a given neural cost associated with the output. We analyzed this proposal in detail for the case that input signals and noise are approximated as Gaussian. Mathematically, the STRF transform can be shown [34] to be composed of three abstract steps: input de-correlation, gain control, and multiplexing. For typical input statistics that are shift-invariant in sound frequency and time, the transform can be compared with two sorts of experimental data. First, gain control corresponds to the magnitude of the modulation transfer function of the STRFs. Second, by choosing the form of multiplexing to arrange the STRFs to have minimal phase, one can predict their full form. That the STRFs or the MTFs adapt to input statistics is a direct prediction of this efficient coding framework, since both the information conveyed and the neural coding cost depend on these statistics. Our efficient coding proposal is thus experimentally testable.

We made two particular predictions about the adaptation of the STRFs, one associated with input intensity, the other with input correlation. For the case of intensity, we predicted that the MTF of the STRFs should become more low pass when input intensity is lowered. Intuitively, as long as inputs at nearby frequencies and times are correlated, a low pass filter smoothes the input to reduce noise, whereas a band pass filter extracts differences between input frequencies and times to remove redundancy. Compared with a band pass STRF, a low pass STRF has one or all of the following characteristics: (1) it has fewer excitatory and inhibitory regions; (2) each excitatory/inhibitory region has a larger size; (3) the secondary or opponent region, e.g., the inhibitory region for a STRF with an primary excitatory region, is weaker. All three characteristics help to smooth noise, a necessary strategy for weak inputs. In contrast, a band-pass filter has the opposite characteristics, so as not to increase the neural cost due to the transmission of redundant input information. These predictions are analogous to those seen in adaptations of visual coding to input SNR [29], [33], [34], [51], [52]. They also generalize previous accounts of the adaptation of the temporal auditory filter [26] to input intensity.

For the case of adaptation to input correlation, our framework predicts that the sizes of the excitatory and inhibitory regions of the STRFs should adapt to the range of input correlations. That is, input ensembles with longer range correlations in frequency and/or time should lead to STRFs with larger excitatory and inhibitory regions in the corresponding feature dimensions. Longer range input correlations are typically equivalent to greater input modulation power in the lower modulation frequency range in the stimulus ensemble. Equally, larger excitatory/inhibitory regions in the STRF are typically equivalent to its MTF being tuned to lower modulation frequencies. Thus, our prediction can be stated equivalently as saying that a stimulus ensemble with greater input power in the lower modulation frequency range, spectrally and/or temporally, should lead to neural MTFs tuned to the lower modulation frequency ranges. We demonstrated this form of adaptation for SRFs in Figure 5, and for STRFs in Figure 7. In particular, with a sufficiently high SNR, the MTF profile 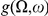 should whiten the ensemble specific input modulation power 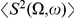.

### Experimental evidence and tests of the predictions

Various experimental observations pertain to these predictions about adaptation to input intensity. Lesica and Grothe [7] presented natural rain sounds to gerbils and found that, for a majority of cells in inferior colliculus (IC), the STRFs have more excitatory/inhibitory regions for higher input sound levels, and only have excitatory regions, or at least very weak inhibitory regions for lower sound levels. Nagel and Doupe [14] conducted a similar study in field L of songbirds, an area analogous to mammalian auditory cortex. In both spectral and temporal dimensions, they found that the excitatory/inhibitory regions of the STRFs become smaller and sharper under higher sound intensity, while the number of such regions do not increase. These results paralleled those of an earlier study in which they only examined the temporal dimension of the receptive fields [58]. Both studies are consistent with our proposal that the MTF changes from lower to higher pass when input intensity (and hence, SNR) increases. They thus offer complementary confirmation of our predictions.

As mentioned in the Introduction, Lesica and Grothe [26] also examined the adaptation of the temporal receptive field(TRF) to vocalizations and ambient noises. They found that the TRF changed from being bandpass to lowpass when noise was mixed into the ensemble of vocalizations, and accounted for this finding in terms of efficient temporal coding. Their result can be understood as a special case of adaptation to SNR in our framework, focusing on the temporal dimension of the STRF, and treating the addition of noise as a reduction in input SNR. According to the principle of efficient coding, the spectral receptive field should also have changed from bandpass to lowpass when this noise was added.

There are as yet few physiological experiments that pertain to our prediction about adaptation to input correlations. One study by Woolley et al [11] examined the STRFs of midbrain neurons in zebra finch in response to bird songs or modulation-limited noise. Compared to that of the noise, the input modulation power of the songs is more concentrated in lower modulation frequencies. The MTFs of the STRFs matched the corresponding modulation frequency spans, consistent with our theoretical prediction.

The studies by Woolley et al [11] and Lesica and Grothe [26] could be extended to different ensembles of natural stimuli, e.g., songs, speech, animal vocalization, and environmental background, each with its own particular input correlations [59]. Findings from such extended studies would provide a stern test of the efficient coding framework. Generally, the input modulation power 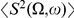 in natural sounds decays with increasing modulation frequency 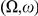, at a rate that is specific to the ensemble [59]. Ensembles with faster decays have longer range input correlations (or larger correlations), as modelled in our Figure 5A and Figure 7BCD. We predict that this decay rate in 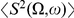 should dictate the shape of the neural MTFs 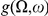, such that ensembles with faster decay should lead to neural MTFs focusing on lower modulation frequency ranges. In particular, for high input SNR, the MTF profile should be that of a whitening filter 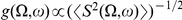, with the upper frequency limit 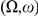 for this whitening (beyond which MTF quickly decays to zero) being around the frequency at which 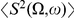 is comparable to the power level of the noise. The recent study by Rodriguez et al [59] showed that inferior colliculus (IC) neurons, when examined collectively as a population, do seem to whiten typical natural stimuli, in that the population MTF 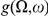 increases with frequency 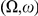 (up to a high frequency limit). This is to be expected for an efficient code, since natural input power 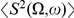 decreases with frequency. However, the neural STRFs in this study were obtained (using the moving ripple stimuli) without specific adaptation to any particular natural stimulus ensemble. We predict that if the STRFs had been measured under adaptation to the natural sounds for high SNR, then the neural MTF profile, at a neural population level if not at individual neuron level, should be ensemble specific, i.e., whitening the input power 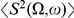 of the adapting stimuli.

### The neural implementation of the efficient STRF and its adaptations

We seek of the overall effective STRF rather than its realization. Thus, it is important to note that the three separate steps of our mathematical analysis of the efficient STRFs are purely abstract. They do not correspond to an actual physiological implementation. In principle, when a receptive field is entirely linear, it can as well be implemented in a single step, as in multiple linear steps in a cascade. Meanwhile, the observation that STRFs adapt to changes in the statistics of auditory inputs, and indeed that visual receptive fields expand when the visual environment changes from bright outdoors to dark indoors [52], attest to the availability of the mechanisms for implementing (and thus adapting) efficient sensory coding.

We speculate that the adaptation of a STRF in a midbrain auditory neuron is likely to involve gain control in many intervening and distributed neural processes upstream along the auditory pathway [60]. Even a simple adaptation of efficient coding, in the large monopolar cells (LMCs) in an insect compound eye to changes in the distribution of input contrasts in the visual environment, involves multiple stages of processes, some in the photoreceptors and others in lamina from the receptors to the LMCs [61]. Synaptic and intrinsic mechanism were also found in the adaptation of retinal bipolar and ganglion cells to temporal contrast [62], [63]. Considering the multiple synapses from the hair cells to IC or auditory cortex, and the many recurrent and feedback networks with both excitatory and inhibitory connections [64], [65] in this pathway (for example, medial olivocochlear (MOC) efferent effects [66]), we speculate that gain control processes are likely to include synaptic facilitation and depression and distributed channel based adaptations. They should collectively achieve the effective adaptation in the gain such as the 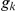 in equation (6) and/or the underlying eigenmodes. Because there are multiple, redundant, and distributed synapses from the auditory periphery to the neuron whose STRF we model, a STRF could be implemented in multiple ways. Such implementational redundancy is likely to be needed to accommodate the many forms of adaptation that might be needed, given a limited degree of flexibility in any individual mechanism.

The timescale of STRF adaptation to sound levels or input SNRs should be less than several or tens of seconds, or even shorter, since, in the physiological experiments, the stimulus duration for one sound intensity level is 40 s in [7] and 5 s in [14], while adaptation to mixing noise into the vocalization inputs occurs within hundreds of milliseconds in [26]. Adaptation has been observed to occur over multiple time scales, ranging from tens of milliseconds to minutes in the fly visual system [67]. In the auditory systems, midbrain neurons adapt to sound levels within hundreds of milliseconds [68], [69], while cortical adaptation happens over multiple timescales and is likely to arise from network activities [70], [71]. We still know too little about the actual mechanisms for STRF adaptation [26] or sensory adaptation in general, although it has been suggested that channel based mechanisms at the cellular level are plausible candidates [67]. Understanding the computational roles of the STRFs should motivate future investigations of these mechanisms.

### Limitations of the framework

As an initial attempt to understand the computational role of the STRFs, our framework has various limitations. First, the STRF model as a whole is quantitatively inaccurate since it specifies a linear mapping between sensory inputs and neural responses (in each adapted state). The accuracy could be improved in future work through the addition of a static nonlinearity after the STRF [6], [7]. However, this would not be expected to lead to a qualitative change in STRFs or their adaptation. Extensions to dynamic nonlinearities would be much more complex. Second, for analytical convenience, we assumed that the input statistics are Gaussian, meaning that there are no input signal correlations higher than second order. The same approximation was made for the case of efficient visual coding, in the absence of good information about higher order input correlations [30], [32], [34]. Subsequent work using independent component analysis (ICA) on natural visual images avoided the Gaussian assumption, leading to models of visual encoding in primary visual cortex V1 [72], [73]. This approach has been adopted to understand the STRFs in the auditory cortex [74] and avian primary auditory area field L [75], although it cannot predict adaptation to SNR and its whitening prediction does not go beyond that obtained under the Gaussian assumption. It is still controversial whether higher order statistics are the cause for the dramatic difference between the V1 encoding and that in the retina and the lateral geniculate nucleus [34]. Furthermore, higher order correlations in natural visual inputs contribute much less redundancy (measured in signal entropy) than second order correlations [36], [37], [38]. This may explain why the Gaussian assumption was not overly deleterious to the predictions of the efficient coding principle in vision. Although higher order correlations in auditory inputs are also poorly understood, they do cause auditory adaptation, e.g., in stimulus-specific adaptation to complex temporal patterns of tones [76]. To what extent higher order input statistics can influence auditory encoding remains to be answered in future studies.

Our focus on coding efficiency ignores aspects of auditory processing devoted to additional tasks such as sound source localization or stream segmentation. The observed STRFs may reflect elements of both efficient coding and requirements associated with these tasks. In fact, some variations are possible within the context of an efficient code. For instance, we have so far restricted ourselves by making all neurons share the same MTF profile predicted by efficient coding (by restricting the 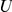 transform to that in equation (9)). Relaxing this restriction would allow other STRFs. In particular, different neurons in the coding population could be tuned to different modulation frequency regions within the 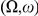 extent covered by the overall MTF envelope 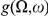, and could have different shapes. Accordingly, different STRFs could have different spectral bandwidths (or resolution) and shapes, in addition to preferring different center frequencies 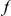. Indeed, in the auditory cortex, different neurons exhibit different spectral resolutions, and even prefer different motion directions of the spectral ripples [77], [78], [19]. (Analogously, primary visual cortical neurons are tuned to multiple spatial sizes and prefer different orientations, a coding scheme that can be shown to be consistent with efficient coding [36].) Such a collection of STRFs could satisfy the joint goals of coding efficiency and detecting ecologically meaningful auditory objects (such as vocalizations). Diversity in the shape and bandwidth of the STRFs is already present, although perhaps less so, sub-cortically, e.g., in inferior colliculus [78]. When different neurons have different STRF bandwidths, our prediction that the input modulation power will be whitened by the neural MTFs should be modified, such that the ‘neural MTFs’ should mean the collective MTF of the whole neural population within a particular auditory stage (such as IC, see [59]).

There could be alternative formulations (other than equation (4)) of the efficient coding principle, in particular, in the formulation of the neural cost. Our formulation 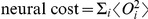 causes the degeneracy of the efficient coding solution, i.e., the existence of many choices of the equally efficient coding transforms, when the signals are gaussian. Other formulations of the neural cost could break this degeneracy. For example, formulation 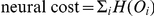 in terms of the summation of individual neural channel capacity (or entropy 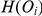), or 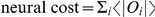 in terms of the total activity level, would generate neural codes to encourage very different MTFs for different neurons. In both audition and vision, the MTFs (in audition) and the contrast sensitivity functions (the vision analog of the MTFs) for different neurons tend to be similar in the sensory periphery (cochlear nucleus and retina), but they are increasingly disparate further towards the central brain. These changes could be caused by the different cost functions in the nervous system, or, as discussed in the previous paragraph, due to the breaking of the degeneracy by additional computational tasks further downstream along the sensory pathway.

Redundancy redunction and information preservation are two essential ingredients of the efficient coding principle. While this principle has been quite successful in understanding the retinal coding, it cannot explain the enormous increase in the redundancy of the visual coding in the primary visual cortex (in which the number of neurons are about 100 times as many as those in the retina) [34], nor the drastic loss of visual information outside the focus of attention in the higher visual areas without introducing task-dependent factors. It remains to be investigated how much and in what form the efficient coding will take further along the auditory pathway. One can expect that more processes will be devoted to solving specific auditory tasks, in addition to the task of sensory encoding, in the higher stages of auditory processing.

### Concluding remarks

This study was partly inspired by the success of the efficient coding principle in understanding receptive fields in the early stages of visual processing, and the way these receptive fields adapt across sensory environments. Analogies between visual and auditory processes have been explored by previous researchers [79], and we expect that they can be carried further in higher level sensory processes including segmentation, selective attention [80], and even object recognition.

In conclusion, efficient coding provides a plausible computational interpretation of various recent experimental observations on STRFs, and notably the way they adapt to input environments. By making testable predictions, it motivates experimental directions which should hopefully lead to further insights and understanding.
